# Mining Patients' Narratives in Social Media for Pharmacovigilance: Adverse Effects and Misuse of Methylphenidate

**DOI:** 10.3389/fphar.2018.00541

**Published:** 2018-05-24

**Authors:** Xiaoyi Chen, Carole Faviez, Stéphane Schuck, Agnès Lillo-Le-Louët, Nathalie Texier, Badisse Dahamna, Charles Huot, Pierre Foulquié, Suzanne Pereira, Vincent Leroux, Pierre Karapetiantz, Armelle Guenegou-Arnoux, Sandrine Katsahian, Cédric Bousquet, Anita Burgun

**Affiliations:** ^1^UMRS 1138, équipe 22, Institut National de la Santé et de la Recherche Médicale, Centre de Recherche des Cordeliers, Université Paris Descartes, Paris, France; ^2^Kappa Santé, Paris, France; ^3^Centre Régional de Pharmacovigilance, Hôpital Européen Georges-Pompidou, AP-HP, Paris, France; ^4^Service d'Informatique Biomédicale, Centre Hospitalier Universitaire de Rouen, Rouen, France; ^5^Laboratoire d'Informatique, du Traitement de l'Information et des Systèmes-TIBS EA 4108, Rouen, France; ^6^Expert System, Paris, France; ^7^Vidal, Issy Les Moulineaux, France; ^8^Institut de Santé Urbaine, Saint-Maurice, France; ^9^Département d'Informatique Médicale, Hôpital Européen Georges Pompidou, Paris, France; ^10^Sorbonne Université, Inserm, université Paris 13, Laboratoire d'informatique médicale et d'ingénierie des connaissances en e-santé, LIMICS, Paris, France

**Keywords:** pharmacovigilance, social media, methylphenidate, drug-related side effects and adverse reactions, drug misuse, data mining, natural language processing

## Abstract

**Background:** The Food and Drug Administration (FDA) in the United States and the European Medicines Agency (EMA) have recognized social media as a new data source to strengthen their activities regarding drug safety.

**Objective:** Our objective in the ADR-PRISM project was to provide text mining and visualization tools to explore a corpus of posts extracted from social media. We evaluated this approach on a corpus of 21 million posts from five patient forums, and conducted a qualitative analysis of the data available on methylphenidate in this corpus.

**Methods:** We applied text mining methods based on named entity recognition and relation extraction in the corpus, followed by signal detection using proportional reporting ratio (PRR). We also used topic modeling based on the Correlated Topic Model to obtain the list of the matics in the corpus and classify the messages based on their topics.

**Results:** We automatically identified 3443 posts about methylphenidate published between 2007 and 2016, among which 61 adverse drug reactions (ADR) were automatically detected. Two pharmacovigilance experts evaluated manually the quality of automatic identification, and a f-measure of 0.57 was reached. Patient's reports were mainly neuro-psychiatric effects. Applying PRR, 67% of the ADRs were signals, including most of the neuro-psychiatric symptoms but also palpitations. Topic modeling showed that the most represented topics were related to *Childhood and Treatment initiation*, but also *Side effects*. Cases of misuse were also identified in this corpus, including recreational use and abuse.

**Conclusion:** Named entity recognition combined with signal detection and topic modeling have demonstrated their complementarity in mining social media data. An in-depth analysis focused on methylphenidate showed that this approach was able to detect potential signals and to provide better understanding of patients' behaviors regarding drugs, including misuse.

## Introduction

Patients use social media to seek information, to receive advice and support from other Internet users in order to better manage their own health care and improve their quality of life (Lamas et al., 2016). Patients and their family share information about drugs in social media; they report on the outcomes and the impact of the drugs on their health and day-life; they describe their attitudes toward the drugs, including adherence to the treatment, adverse events and sentiment (Laranjo et al., 2015). Consequently, social media data mining has been recognized by drug agencies as a potential approach to identify patient reporting of adverse drug reactions (ADR), and to analyze the attitudes and knowledge of general public and patients on medicines. The Food and Drug Administration (FDA)^1^ in the United States and the European Medicines Agency (EMA)^2^ are considering social media as a new data source to strengthen their surveillance activities. Several authors have compared traditional data sources and social media. They demonstrated similarities between these sources to detect signals about adverse reactions but suggested that social media sources contained different information (such as less serious events and more adverse effects related to their quality of life) and were used by patients. As healthcare professionals mainly report to drug agencies, social media may be a complementary source about drug use and safety. This conclusion was shared by, e.g., Duh et al. who analyzed posts related to atorvastatin, a lipid-lowering agent and sibutramine, an appetite suppressan drug (Duh et al., 2016), and by Pages et al. who focused on oral antineoplastic drugs (Pages et al., 2014). However, we still lack a deep understanding of the characteristics of patient reported information about ADRs and patient attitudes regarding drug therapies, which hinders clear guidance on how to adapt text mining tools for social media and how to use them for decision in public health and drug safety (Golder et al., 2015). Dedicated tools may help experts to extract relevant information from the data available in different sources without spending time to explore manually the data (Lardon et al., 2015; Nikfarjam et al., 2015; Sloane et al., 2015). Nevertheless, Sarker et al. showed that the most popular algorithms in the published studies were supervised classification techniques to detect posts containing ADR mentions, and lexicon-based approaches to extract named entities from texts (Sarker et al., 2015). We believe that such approach still requires enhancements to support data mining in pharmacovigilance, particularly in making the data generated by patients more prominent, explicit, and accessible to experts in pharmacovigilance. Moreover, data mining should not be limited to ADR detection but rather integrate all kinds of information reported in posts, like patients' attitudes toward the treatment, compliance, and misusage.

The main objectives of the ADR-PRISM project (Bousquet et al., 2017) were to perform text mining and visualization tools to enhance our understanding of patient reported information in social media and to assess how it could be used for pharmacovigilance purpose. In the context of ADR-PRISM, we conducted an in-depth content analysis of the anonymized data publicly available on methylphenidate on five open French patient forums. This study is used in this article to present the methods that we developed to mine social media and to illustrate their results. The research done in the framework of ADR-PRISM has been supported by an Ethics Advisor Board. The Ethics Advisory Board was composed of scientists with different scientific backgrounds: Gaby Danan from Pharmacovigilance Consultancy; Alain-Jacques Valleron from INSERM UMR1169 and Paul-Olivier Gibert from Digital&Ethics, and provided independent advice on the project.

## Background

### Pharmacovigilance

Drug safety, also referred as pharmacovigilance, focuses primarily on ADRs, which are defined as “a response to a drug which is noxious and unintended^3^.” It also encompasses medication errors, misuse, overdose and abuse (World Health Organization, 1972). The spontaneous reporting system is widely used and effective for pharmacovigilance, and its major limitation is under-reporting. Hazell and Shakir estimated a median under-reporting rate based on 37 published studies as high as 94% (interquartile range 82–98%) (Hazell and Shakir, 2006). Reasons that could explain under-reporting are numerous: (i) frequent minor events like headache are less likely to be reported by health professionals; (ii) health professionals may not find it necessary to report the events that are very frequent thus expected (iii) even when the symptoms are severe, they may not be recognized as a possible ADR; for example, Hazell and Shakir found the median under-reporting rate for serious or severe ADRs was still very high (95%).

Under-reporting of ADRs by patients in spontaneous reporting systems like the FDA Adverse Event Reporting System (FAERS) has also been observed, with only 20–33% of the minimum number of expected serious events being reported (Alatawi and Hansen, 2017). Consequently, several authors have reached to the conclusion that social media listening is an important tool to augment post-marketing safety surveillance (Powell et al., 2016; Koutkias et al., 2017). However, these authors consider that much work is needed to determine the best methods for using this data source. Besides ADRs, the messages in social media can be used to explore other behaviors related to pharmaceutical treatment, like non-compliance, misuse, overdose, and abuse that have to be studied in real life contexts. In the rest of this article, we will consider “misuse” in its broader meaning, which encompasses the definition provided by the World Health Organization (WHO), i.e., the use of a substance for a purpose non consistent with legal or medical guidelines (like nonmedical use), and the FDA's one i.e., off-label use (Anderson et al., 2017).

### Methylphenidate

Attention deficit hyperactivity disorder (ADHD) is a highly prevalent disorder in many countries, with an estimated prevalence of 5–10% in children worldwide (Lee et al., 2007). Methylphenidate is a psychostimulant primarily marketed under the name of Ritalin®, whose first marketing authorization was given in France in 1995 for ADHD in children aged 6 years or over. Methylphenidate is nowadays broadly used in many countries. The literature associated with methylphenidate is abundant (a Medline query on Nov 14th, 2017 with methylphenidate as keyword retrieved more than 10,000 articles). We will only provide a broad view of the results published recently. Over the last two decades, the use of ADHD medication in US youth has markedly increased. More than 1.5 million US adults use stimulants labeled for treatment of ADHD (Habel et al., 2011) and more than 2.7 million children are prescribed medications to treat ADHD in the U.S. each year, with an increase from 3.3 to 3.7% (+10.7%) between 2005 and 2012. In Europe, a repeated cross-sectional design applied to national or regional data extracts from Denmark, Germany, the Netherlands, the United Kingdom (UK) showed significant increase of ADHD medication prevalence in the same period with discrepancies across countries: from 1.8 to 3.9% in the Netherlands (relative increase: +111.9%), from 1.3 to 2.2% in Germany (+62.4%), from 0.4 to 1.5% in Denmark (+302.7%), and from 0.3 to 0.5% in the UK (+56.6%) (Bachmann et al., 2017). The prescription in France of methylphenidate remains very limited compared to that of other European countries or North America. The French Agency for Drug and Health product Safety (ANSM) estimated that around 49,000 patients (regardless of their age) were given methylphenidate in France in 2014, most of them being children between 6 and 11 and teenagers from 12 to 17^4^. Adherence to treatment is a significant issue, since 61% of adolescents who were prescribed methylphenidate reported being non-adherent to their treatment (Kosse et al., 2017).

Concerns have been expressed about possible cardiac effects of methylphenidate, regarding its pharmacological characteristics and the first post-marketing data (Awudu and Besag, 2014). Increase in mean heart rate and blood pressure have been reported, although most of the studies have not yielded statistically significant results (Cooper et al., 2011). Decreased appetite and sleeping disorders have been reported (Kosse et al., 2017).

Furthermore, concerns about off-label use and abuse of methylphenidate have been expressed by drug surveillance agencies. In 2009, the EMA requested for further studies regarding the use of methylphenidate in Europe; in a report published in 2013^5^, the French Agency (ANSM) described the use of methylphenidate by non-ADHD patients, based on the data collected by the national health insurance on one hand, and by the French network for Pharmacodependence on the other hand^6^. Methylphenidate was prescribed to treat sleeping disorders, anxio-depressive disorders, agitation, and was also used for cocaine substitution, weight loss and doping (Kosse et al., 2017). The non-medical use of prescription stimulants like methylphenidate has become the subject of great interest for its diffusion among university students. This phenomenon has been widely investigated in the U.S. due to its increasing trend (Weyandt et al., 2016). Recent research reported the prevalence rate of stimulant misuse was estimated to range between 13 and 23%, approximating around 17% on average (Benson et al., 2015). In similar studies conducted in Europe (Dietz et al., 2013; Deline et al., 2014), findings generally reflect those from the United States. As a recent example, 11.3% of university students in a Northern Italian geographic area reported non-medical use of prescription stimulants (Majori et al., 2017). Studies consistently indicate that the main reasons to use them is cognitive and academic enhancement (Benson et al., 2015; Weyandt et al., 2016), and—to a lower extent—sports performance (Majori et al., 2017). Moreover, route of administration affects the potential effects of methylphenidate. When methylphenidate is abused intra-nasally, the effects are similar to intranasal use of amphetamines and cocaine. Exposure to excessive doses of methylphenidate could increase the risk of serious adverse cardiovascular and psychiatric effects.

Key points regarding drug safety for methylphenidate can be summarized as follows: (i) it is an important treatment option for ADHD patients, with an increasing number of patients being prescribed this medication, especially children or young adults; (ii) there are concerns about adverse effects of methylphenidate in patients that use it regularly to treat ADHD, with a particular concern about long-term use; (iii) it has potential for misuse and abuse. Detecting adverse events, misuse and abuse is a difficult challenge, and this task will benefit from mining of narratives about methylphenidate available in social media.

### Text mining

Analysis of a huge number of narrative messages requires text mining techniques. Information extraction is one of the typical text mining tasks, whose goal is to create a structured view of the information presented in human language text, and to make it more accessible for machine processing. An initial processing step is Named entity recognition (NER), which involves identifying in text instances of predefined categories. Early work in NER systems in the 1990s was aimed primarily at extraction of people and location names from journalistic articles. Very quickly NER has been considered in molecular biology and bioinformatics domain to identify genes and gene products. Significant effort is also spent on extracting chemical entities and drug names in the context of the CHEMDNER competition (Krallinger et al., 2015) for automatic retrieval from biomedical documents. Once the named entities have been identified, the subsequent processing step is identification of semantic relationships between the entities, for example, protein-protein interactions in bioinformatics (Blaschke et al., 1999), and chemical-disease relations in biomedical domain (Wei et al., 2016).

For drug safety monitoring, information extraction is an essential tool to extract both drug names and adverse reaction description, and then to identify if there is a causal relationship between them. Various studies have looked at extracting potential ADRs from different text sources (Yeleswarapu et al., 2014), which include Electronic Health Records (EHR) (Wang et al., 2009; Luo et al., 2017), medical case reports (Gurulingappa et al., 2012) and MEDLINE abstract (Avillach et al., 2013). More challenges have appeared when considering using texts from social media due to the informal and colloquial expressions of internet users (Liu and Chen, 2015).

Topic modeling is another promising text mining approach, which aims to discover hidden semantic structures in a collection of texts. Topic models constitute a set of probabilistic models allowing to explore, understand and organize large groups of structured or unstructured data (Blei et al., 2003). This family of models is based on the hypothesis that documents in the corpus correspond to a distribution of several topics. No prior assumption is made about the nature of topics pervaded in the studied corpus. The outcome of such models is twofold: (i) the list of thematic in the corpus, (ii) the distribution of topics on documents, that enables the clustering of similar documents. By determining discussed topics, topic models provide an automated process for classifying, organizing and managing messages. This process can provide a compact description of the corpus of messages without human intervention. The simplest form of this kind of models is the Latent Dirichlet Allocation (LDA) (Blei and Lafferty, 2009).

Topic models have been used to analyze messages on social media in several domains with promising results. Most of the studies have focused on tweets (Paul and Dredze, 2011, 2014; Prier et al., 2011; Ghosh and Guha, 2013; Zhan et al., 2017). Regarding forum messages, this approach has been used to analyze different behavioral health challenges (Yesha and Gangopadhyay, 2015), to investigate social support (Wang et al., 2012; Portier et al., 2013), topics that health consumers discuss when reviewing their health providers online (Brody and Elhadad, 2010; Hao and Zhang, 2016; Hao et al., 2017), and quality of life of breast cancer patients (Tapi Nzali et al., 2017).

Topic modeling has also been proven useful to detect messages reporting ADRs. Yang et al. applied the LDA model to represent posts in the topic space and were able to extract topics related to ADRs like (for example) diarrhea for Biaxin (Yang et al., 2015). A model using Labeled Latent Dirichlet Allocation (LLDA) (Ramage et al., 2009) exhibited good performance in extracting ADRs from forum posts (Yates et al., 2015). Recently, we applied LDA to detect posts describing non-adherence to drug treatment and tested this approach with posts related to escitalopram (an antidepressant drug), and aripiprazole (an antipsychotic drug) with encouraging results.

In the study presented in this article, we decided to use the Correlated Topic Model (CTM) (Blei and Lafferty, 2006) to investigate misuse of methylphenidate in forum discussions. Besides better fitting text corpora, this model takes into account existing relations between discussed topics. Correlations are estimated by replacing the prior Dirichlet distribution by a logistic normal prior. Estimated correlations between topics indicate to what extent some themes appeared simultaneously in posts. To our knowledge, this is the first study applying the CTM to health related forum posts.

In the following sections, we present two automated methods tailored for drug safety based on social media: (i) signal detection based on text mining techniques. (ii) an in-depth exploratory and qualitative analysis of the data related to methylphenidate. Finally, the benefits and limitations of the approach are discussed.

## Materials and methods

### Material

#### Corpus

Messages were collected using the Detec't extractor, a scraper developed by Kappa Santé. We selected five popular and open French forums: www.atoute.org, www.doctissimo.fr, www.e-sante.fr, www.onmeda.fr (previously www.aufeminin.com) and sante-medecine.journaldesfemmes.com according to their popularity and their quality evaluated by the Net scoring tool (Katsahian et al., 2015). The extraction was based on a set of 403 drugs from the French Health Insurance database used as keywords to extract the corpus of messages. All data extracted was publicly available and anonymized. We identified all the messages containing these drug names and extracted the whole discussions containing these messages. To extract methylphenidate related data, we used the following French brand names of the drug: Ritaline®, Quasym®, Concerta® and Medikinet®. The methylphenidate sub corpus contained all posts where at least one of these drug names was present.

Scraping of these messages was performed according to the HTML structure of each forum. Posts from the retrieved discussions were stored in the Detec't database with all their metadata (date, author of the post, post ID, URL, name of the forum) and cleaned (removing of ads, and quotation from other patients).

#### Medical thesauri

Three thesauri were used to represent the medical terms: RacinePharma, the Anatomical Therapeutic Chemical (ATC) classification system for drugs^7^, and the Medical Dictionary of Regulatory Activities (MedDRA) for disorders^8^. RacinePharma was used to identify drug names in the messages. This resource is updated on a monthly basis to follow the modifications in the French Public Database of Medications^9^. Rationale for choosing RacinePharma is that it covers all medications available on the French market. All commercial drug names were mapped to their active substance, allowing for grouping drugs that have the same active substance. ATC provides a hierarchy of drugs with five levels: (1) anatomical main group, (2) therapeutic subgroup, (3) pharmacological subgroup, (4) chemical subgroup and (5) chemical substance. MedDRA was used to identify medical terms including symptoms, signs, diseases, diagnoses, names and results of analysis etc. MedDRA has a hierarchical structure of five levels: (1) SOC (system organ class), (2) HLGT (high level group terms), (3) HLT (high level terms), (4) PT (preferred terms) and (5) LLT (lowest level terms), which contains lexical information like synonyms, lexical variants, lay terms etc. Annotation of the ADRs was done using the finest *chemical substance* level in ATC and the LLT level in MedDRA. When assessing the signals, we decided to consider the PT level, which groups all synonyms that might be used by different reporters.

### Descriptive statistics

Descriptive statistics were used to provide first insights on the characteristics of the corpus (number of messages, origins of messages, etc.). For the methylphenidate sub corpus, the trends regarding the numbers of messages across time were analyzed along with the pharmacovigilance key events related to this drug. A wordcloud, which is a visual representation of the words present in a corpus, where the size of the words represents their frequency in the corpus, was produced to identify the most frequent words in the methylphenidate sub corpus.

### Signal detection

#### Identifying ADRs in posts

This module comprises two steps: the first one consists in recognizing drug names and medical concepts in text using NER methods; the second one consists in identifying the semantic relation between these entities, i.e., the ADR relation. The Smart Taxonomy Facilitator (STF) Skill Cartridge™ developed by Expert System was applied on the corpus for the NER task. It combines a rule-based approach and a dictionary-based approach. The latter includes two main technologies, (i) Fuzzy Term Matching, which takes into account possible variants of the terms present in the taxonomy, thus reducing the number of false negatives, (ii) Relevance Scoring, which applies a series of heuristics that assign a score to each extracted concept, thus eliminates the least relevant concepts in order to reduce false positives. STF also exploits lexical labels (part-of-speech tagging) to address ambiguity issues.

An ADR may be represented as a ternary relationship involving a patient, a drug and a symptom related with this drug through a causal relationship. In addition to causal relation linguistic patterns that corresponded to five major semantic relations between these three entities have been identified: (1) administration (take, test, try, treatment, intake of, etc.), (2) causal relationship (cause, give, result of, since, because of, etc.), (3) sensation (suffer, feel, etc.), (4) interruption of treatment (stop to avoid, to arrest, etc.) and (5) intolerance (endure, allergy, etc.). With the pre-defined linguistic patterns, we were able to identify multiple relationships between drugs and symptoms within one sentence.

#### Statistic models for signal detection

Signal detection is based on statistical measures of association describing reporting disproportionality. If the statistical measure crosses certain threshold, which is summarized as a decision rule, then signal is declared for a given drug associated with a given symptom. Evans et al. used Proportional Reporting Ratio (PRR) to measure the disproportion (Evans et al., 2001). Rothman et al. improved PRR with Reporting Odds Ratio (ROR) (Rothman et al., 2004). Bates et al. developed Bayesian Confidence Propagation Neural Network (BCPNN) model which considered the information component of drug-ADR combinations (Bate et al., 1998), and DuMouchel developed Gamma Poisson Shrinker (GPS) model using Empirical Bayes Screening to quantify disproportion (DuMouchel, 1999). All these methods have been evaluated in a number of empirical studies as well as in several comparative simulation studies (van Puijenbroek et al., 2002; Roux et al., 2005; Ahmed et al., 2010) as broadly comparable, and have been used by different regulatory agencies and drug safety monitoring systems.

We applied all these four methods to our database and listed the signals detected with each method in Table 5. Our results showed that more signals were detected with the two frequentist methods. As the objective of this work was not to evaluate different signal detection methods, we limited ourselves to analyze the signals obtained for methylphenidate with PRR and then compared them with two other sources: (i) the adverse effects described in the Summary of Product Characteristics (SPC) of methylphenidate and (ii) the suspected ADRs reported in Vigibase^10^, which is the WHO's global database for ADRs filled with Individual Case Safety Reports (ICSRs) collected in over 110 countries and spans over more than 100,000 different medicinal products.

The automated annotations were parsed with R 3.3.1 xml2 package, and the signal detection was performed using PhViD R package.

### Topic model

#### Preliminary data processing for topic model

Preliminary data processing was performed for cleaning and formatting the methylphenidate sub corpus so that the topic model can be applied on it and text categorization can be achieved. To this purpose, the corpus was transformed into a matrix where each line represents a message and each column represents a token (i.e., a term present in the corpus). This matrix is called the document-term matrix (DTM).

Data cleaning was performed as follows:

1. Preprocessing: All words in messages were turned into lowercases. French accents, punctuation and abusive spaces between words were removed.
2. Removal of stopwords: In addition to stopwords, the names of the drug in the messages (e.g., Ritaline® or methylphenidate) were excluded since this information was already taken into account as message annotations.
3. Addition of specific tags: Two types of tags were added to standardize the mentions of doses (numeric characters followed by “mg” were replaced by the tag “dosemilligrams”) and of duration (using the tags “nbdays,” “nbweeks,” “nbmonths” and “nbyears”).
4. Stemming, based on the Porter's algorithm, was performed to associate inflected and derived words together with their root form.

The next phase, formatting, aimed at generating the final matrix:

1. The list of tokens was created. Tokens were words or sequences of two words (bigrams) found in the corpus. Rationale for considering bigrams was to keep nominal groups like “side effect” (“effet indésirable” in French). The frequency of each token in the messages was measured.
2. The DTM was then created. Due to important discrepancies in the vocabulary used in messages, a vast majority of tokens was associated with a very low number of occurrences in messages.
3. To exclude these words hardly used (including words with spelling errors), the words present in only a small number of messages were removed. The threshold was determined empirically.
4. Finally, we applied DTM weighting, based on *term frequency inverse document frequency* (tf-idf) (Salton and McGill, 1986). Each term in the DTM was weighted according to its frequency in the document and in the entire corpus.

#### Probabilistic models for topic estimation

In order to determine discussed topics in the methylphenidate sub corpus and to identify the associated messages, a topic model was applied on the weighted DTM. We used the Correlated Topic Model (CTM) because (i) it is based on the Latent Dirichlet allocation (LDA), which has been proven providing better semantic coherence and interpretability (Stevens et al., 2012); (ii) it takes into account existing relations between discussed topics as an additional parameter. Estimated correlations between topics indicate to what extent some themes appeared simultaneously in posts, indicating this way which themes are associated. The number of topics was determined by choosing the value maximizing the log-Bayes Factor (Taddy, 2012).

The model was estimated using a Variational Expectation Maximization (VEM) algorithm. As previously mentioned, the modeled topics are probability distributions over the words found in the corpus. To determine each topic, words were ranked from highest to lowest tf-idf value of their probability in this topic (Blei and Lafferty, 2009). For each topic, the first 15 words were designated as the set of characteristic words and used to interpret its semantics.

We applied the method described above to the formatted methylphenidate sub corpus, and focused on the topics related to ADR, misuse and abuse. The posts related to these topics of interest were analyzed thanks to descending hierarchical classification (DHC) on observed words. The analyses were performed using the STM (Structural Topic Model) package (Roberts et al., 2014) with the R software. The DHC was performed using the software Iramuteq and the ALCESTE classification.

## Results

### Dataset

#### Overall corpus

Twenty one million messages have been extracted. The messages were all posted between the 1st January 2007 and the 31st January 2016. This data set was used as a basis for signal detection. Based on the automatic annotation process described above, we have identified 31,586 ADRs, concerning 1,426 distinct drug names (representing 1,055 unique ATC codes) and 1,775 distinct symptoms (representing 1,154 unique MedDRA PTs). The five most common chemical substances involved were paracetamol, clomifene, venlafaxine, plastic IUD with progestogen and levothyroxine sodium (Table 1), the five most common PTs involved were pain, weight increased, fatigue, nausea and acne (Table 2).

**Table 1:** Top 10 chemical substances with ADRs on French forums between 2007 and 2016.

**ATC code**	**Name**	**Occurrences**
N02BE01	Paracetamol	840
G03GB02	Clomifene	838
N06AX16	Venlafaxine	761
G02BA03	Intrauterine contraceptive device with progestogen	735
H03AA01	Levothyroxine sodium	669
N06AB10	Escitalopram	581
G03HB01	Cyproterone and estrogen	546
A03AX12	Phloroglucinol	538
N05AX12	Aripiprazole	504
N06AB05	Paroxetine	428

**Table 2:** Top 10 commonly discussed symptoms with ADRs on French forums between 2007 and 2016.

**MedDRA PT**	**Occurrences**
Pain	2,417
Weight increased	1,425
Fatigue	986
Nausea	903
Acne	784
Convulsion	682
Malaise	654
Anxiety	637
Insomnia	604
Headache	597

#### Sub-corpus of messages related to methylphenidate

The methylphenidate sub corpus contained all messages belonging to the Detec't database published between the 1st January 2007 and the 31st January 2016 containing at least one of the following French brand names: Ritaline®, Concerta®, Quasym® and Medikinet®. This corpus contained 3443 messages from five different sources, with 75% of messages coming from Doctissimo (Table 3). Ritaline® was the most frequent brand name in the sub-corpus (Table 4).

**Table 3:** Messages distribution by forum.

**Forum**	**Number of messages**	**Number of annotations**
Atoute	631	1,134
Doctissimo	2,569	4,337
E-sante	227	439
Onmeda	5	12
Sante Medecines	11	12

**Table 4:** Methylphenidate brand names distribution.

**Forum**	**Number of annotations**
Ritaline®	4,256
Concerta®	1,379
Quasym®	275
Medikinet®	24

##### Trends in the methyphenidate sub-corpus

**Figure 1:**
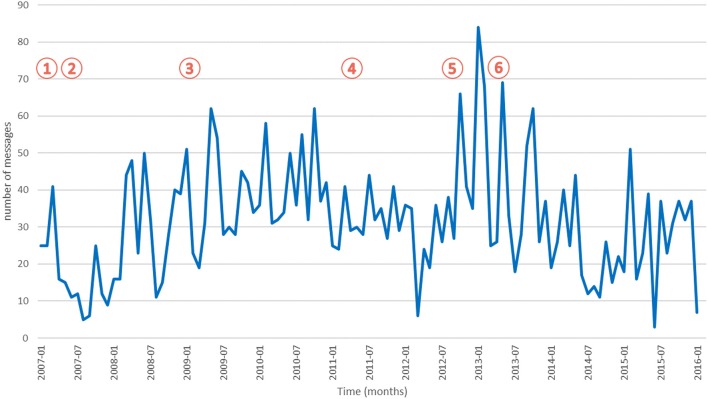
Number of methylphenidate messages across time. Indexes of key events are indicated inside red circles.

The distribution of messages per month is displayed in Figure 1. Methylphenidate use has been in constant increase since 2004. We identified six main events related to drug safety and methylphenidate during this period:

1. In 2006, the ANSM (formerly Afssaps) launched a new national initiative for pharmacovigilance and addictovigilance monitoring;
2. In 2007, the EMA initiated a review of the safety of methylphenidate;
3. In January 2009, the EMA experts stated that the benefits of methylphenidate continued to outweigh their risks, when they were used in their approved indication. However, they insisted on the needs for vigilance regarding long term risks;
4. In June 2011, the French pharmacovigilance published a warning letter regarding possible long term effects of medicines containing methylphenidate;
5. In October 2012, the French National Authority for Health (HAS) published a directive reassessing medicines containing methylphenidate. Their conclusion was that uncertainties still existed on medium to long term effects, in particular for cardiovascular, neurologic and psychiatric effects, and that there was a risk of non-medical use, misuse and abuse;
6. In July 2013, the ANSM published a report toward health professionals based on a review of methylphenidate use in France in order to secure methylphenidate prescription. Concomitantly, they published a brochure specifically dedicated to consumers to inform the patients and their families.

The comparison of key events and trends in messages did not reveal any association between those two phenomena, except for the HAS directive in 2012 (event #5), which was associated with a higher number of forum discussions related to methylphenidate in the following months.

##### Word cloud of messages with presence/take of methylphenidate

**Figure 2:**
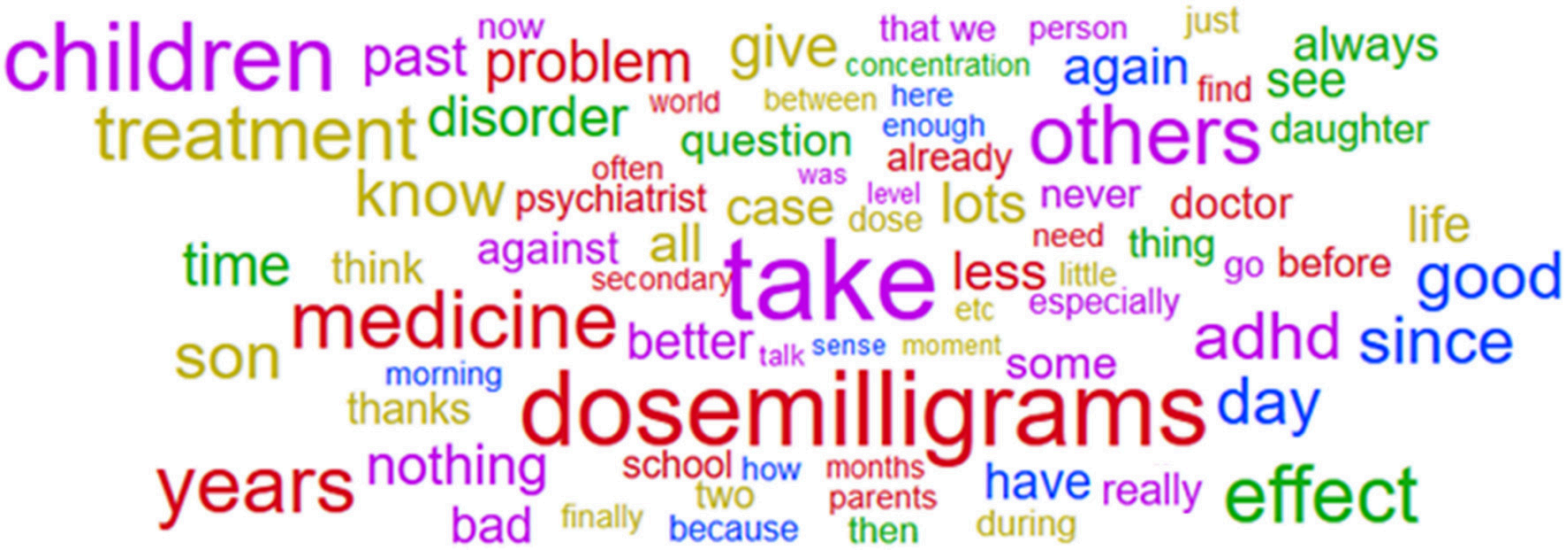
Wordcloud on the methylphenidate corpus.

First clues of topics discussed in the corpus were given by the analysis of the wordcloud. In Figure 2 are displayed the most frequent words present in the methylphenidate corpus after translation from French to English. Starting from the 100 most frequent words, translation from French to English led to a total of 77 distinct English words (for example “*take”* corresponds to both “*prends”* and “*prendre”*). The most frequent words were “*take,” “children,”* and “*dosemilligrams.”* As mentioned before, the term “*dosemilligrams”* was used as a tag to replace and standardize the mention of dose in all the messages. Several lexical fields are represented in the methylphenidate wordcloud such as ADHD (“*ADHD,” “disorder,” “concentration”*), drug prescription and intake (“*psychiatrist,” “doctor,” “take,” “medicine,” “treatment,” “dosemilligrams”*), childhood (“*children,” “son,” “daughter,” “school,” “parents”*) and concerns about adverse effects (“*question,” “problems,” “effect,” “secondary”*). No specific side effect could be observed at this point, although some words associated with this topic (like “*effect,” “secondary,” “bad”* and “*problem”*) were present in many discussions. The wordcloud showed a high frequency of words related to the use of methylphenidate by children and consistent with the primary indication of the drug. No hints for misuse could be identified at this point.

### Detected ADRs and signals

We identified 61 ADRs associated to methylphenidate with causal relationship in the corpus, which corresponded to 39 distinct effects and eight MedDRA SOC level terms (Table 5), upon the 3,443 messages with methylphenidate mention. The most common effects were psychiatric effects (25 cases, 41%) and nervous system disorders (19 cases, 31%). Weight loss was also mentioned, and one case of cardiac side effect was reported. Side effects of SOC cardiac disorders and nervous system disorders were considered of particular interest, since the ANSM expressed concerns about cardiac effects (hypertension, heart rhythm disorders) and neurological adverse events (migraine, stroke) in 2011 and 2012.

**Table 5:** Comparison of detected ADRs of methylphenidate with SPC and Vigibase.

	**SOC**	**Frequency**	**Detected as a signal**				**Described in SPC**	**Reported in VigiBase**
	PT		**PRR**	**ROR**	**BCPNN**	**MGPS**		
	**Psychiatric disorders**	**22**					
1	Aggression	3	x	x	x	x		x
2	Psychotic disorder	3	x	x	x	x		x
3	Anger	2	x	x	x	x		x
4	Anxiety	2				x	x
5	Abnormal behavior	1	x	x			x
6	Affective disorder	1	x	x			x
7	Delusion	1	x	x			x
8	Dependence	1				x	x
9	Depression	1				x	x
10	Euphoric mood	1					x
11	Negativism	1					x
12	Nervousness	1	x	x		x	x
13	Paranoia	1	x	x			x
14	Sleep disorder	1	x	x		x	x
15	Stress	1					
16	Substance abuse	^1^	x	x			x
	**Nervous system disorders**	**17**					
17	Psychomotor hyperactivity	4	x	x	x	x	x	x
18	Headache	3	x	x		x	x
19	Convulsion	3	x	x		x	x
20	Insomnia	2				x	x
21	Tic	2	x	x	x	x	x	x
22	Dyslexia	1					x
23	Crying	1	x	x			x
24	Poor quality sleep	1	x	x			x
	**Psychiatric disorders, nervous system disorders**	**2**					
25.	Disturbance in attention	2	x	x	x	x		x
	**Investigations**	**8**					
26.	Weight decreased	3	x	x		x	x
27.	Amphetamines	2	x	x	x	x		x
28.	Heart sounds	1	x	x			
29.	Neuropsychological test	1					
30.	Weight	1					
	**General disorders and administration site conditions**	**7**					
31.	Fatigue	3				x	
32.	Malaise	2					
33.	Drug intolerance	1					x
34.	Rebound effect	^1^	x	x			x
	**Psychiatric disorders, general disorders and administration site conditions**	**1**					
35.	Irritability	^1^	x	x		x	x
	**Cardiac disorders**	**1**					
36.	Palpitations	^1^	x	x		x	x
	**Musculoskeletal and connective tissue disorders**	**1**					
37.	Muscle spasms	^1^	x	x		x	
	**Metabolism and nutrition disorders**	**1**					
38.	Decreased appetite	^1^	x	x			x
	**Neoplasms benign, malignant and unspecified (incl cysts and polyps)**	**1**					
39.	Neoplasm malignant	^1^	x	x			
	Total	^61^					

Two pharmacovigilance experts (ALL and CB) manually reviewed the content of these messages. In cases where the same ADRs were mentioned multiple times in the same messages, or where patients posted the same message several times, the duplicates were removed. After removing these duplicates, we obtained 57 relevant cases posted in 46 messages, including 28 messages written by the patient, and 18 posted by a relative, mostly the patient's parents.

61.4% of the ADRs (35 of 57) were validated by the experts, whereas 38.6% (22 of 57) were considered as false positives. For example, in one message the patient talked about his treatment of ADHD, by methyphenidate and his mother having a cancer: cancer was annotated by the system as an ADR, but it was a false positive.

There were 33 additional ADRs in the messages that have not been automatically identified. These ADRs were not easily detectable as the name of the drug was not present in the same sentence as these effects. These missing adverse effects included sleep disorder, weight decrease, gastrointestinal disorder and decreased appetite.

We identified several cases of drug abuse. In one message the mother disclosed taking her son's treatment and experiencing positive effects on her mood; the other case of misuse was about snorting Ritalin. Four patients had incoherent speech probably caused by delusional ideas, which made it impossible to conclude whether the effect was the consequence of taking methylphenidate or the manifestation of a psychiatric disorder. For example, one patient declared that her psychiatrist had prescribed several times the same treatment, using a pictorial expression “multiplication of Ritalins.” Four patients described addictions to other products such as amphetamine or cannabis and four other patients described abusing methylphenidate. Methylphenidate was inefficient in seven patients.

In some cases, the patient perceived the adverse effect as a beneficial effect, for example, one patient considered weight loss and decrease of appetite as a positive effect, and another patient said “it wakes me up and I'm more motivated, it has an antidepressant effect and I had euphoria.”

Considering the signals detected using PRR, the overlap with other data sources was important: 66.7% of the identified relationships (26 of 39) are detected as a signal, among which, 38.5% (10 of 26) have been mentioned in the product SPC, and 88.5% (23 of 26) have been alarmed in VigiBase. We obtained signals for neuro-psychiatric symptoms but also for a cardiac symptom (palpitations). The missing adverse effects (false negatives) could potentially enhance certain signals. Despite some false negatives, sleep disorder and weight decreased have still been detected.

### Topic analysis

#### Overall analysis

At the end of the preprocessing steps, we obtained a DTM containing 1,560 tokens and 3,416 messages. The application of the model identified 14 topics. The topics and their characteristic words (translated in English) are presented in Table 6.

**Table 6:** Topics in the methylphenidate corpus.

**Topic name**	**Characteristics words**
1. Questions	Thanks, would like, testimony, medical, answer, someone, good evening, come, know if, neurologist, since, help, in advance, question, know
2. Positive treatment effects	Adhd, certain, case, person, effectively, a lot, allow, task, when, positive, some, people, shrink, other, pathology
3. ADHD	Child, trouble, add, attention, hyperactive, the hyperactivity, adhd, attention disorder, scolar, hyperactivity, difficult, diagnostic, deficit, attention, result
4. Other products	Withdrawal, medical, cocaine, secondary effect, depression, wellbutrin, anti, effect, secondary, psychiatric, product, rivotril, prescription, sleeping pill, xanax
5. Prescription	Medical, prescription, magnesium, prescribed, prescribe, reembourse, attending physician, attending, product, site, psychiatrist, prescribe, non-specialized, see doctor, adhd
6. Side effects and dosage	Secondary effect, effect, secondary, dose, morning, dosage, effect, strattera, no effect, biphentine, of secondary effect, loss appetite, start treatment, take, evening
7. Negativity and fears	Doc, fear, thing, buy, shrink, skill, impression, awareness, email, interruption, schizophrenic, medicine, schizophrenia, return, state
8. Care pathways (adults)	I was, I had, risperdal, crisis, during, nausea, neurologist, psychiatrist, did not have, did, ocd, fatigue, had, start, interruption
9. Child health care	Child, hyper, son, sister, pedopsychiatrist, house, hyperactive, fright, lesson, cry, clever, kiss, forget, husband, heart
10. Treatment initiation	Medicine, impression, really, good, try, take, add, know if, sensation, coming, good, take, concentrated, get better, should
11. Child history	Son, nbyears, scolar, daughter year, neuropediatrician, home, boy, pedo, treatment, help him/her, since, get better, appointment, years and a half, on since
12. Temporality	Evening, morning, sleep, day, wish, night, sleep, Monday, every, tomorrow, bed, job, well, all alone, hard
13. Studies and papers	Study, disorder, treatment, diagnostic, article, in particular, child, clinical, anti, research, schizophrenic, associated, psychiatric, analysis, answer
14. Non-identified	Awareness, measure, condition, field, skill, alone, of a, question, affect, recognize, sadly, absence, schizophrenic, represent, through

**Figure 3:**
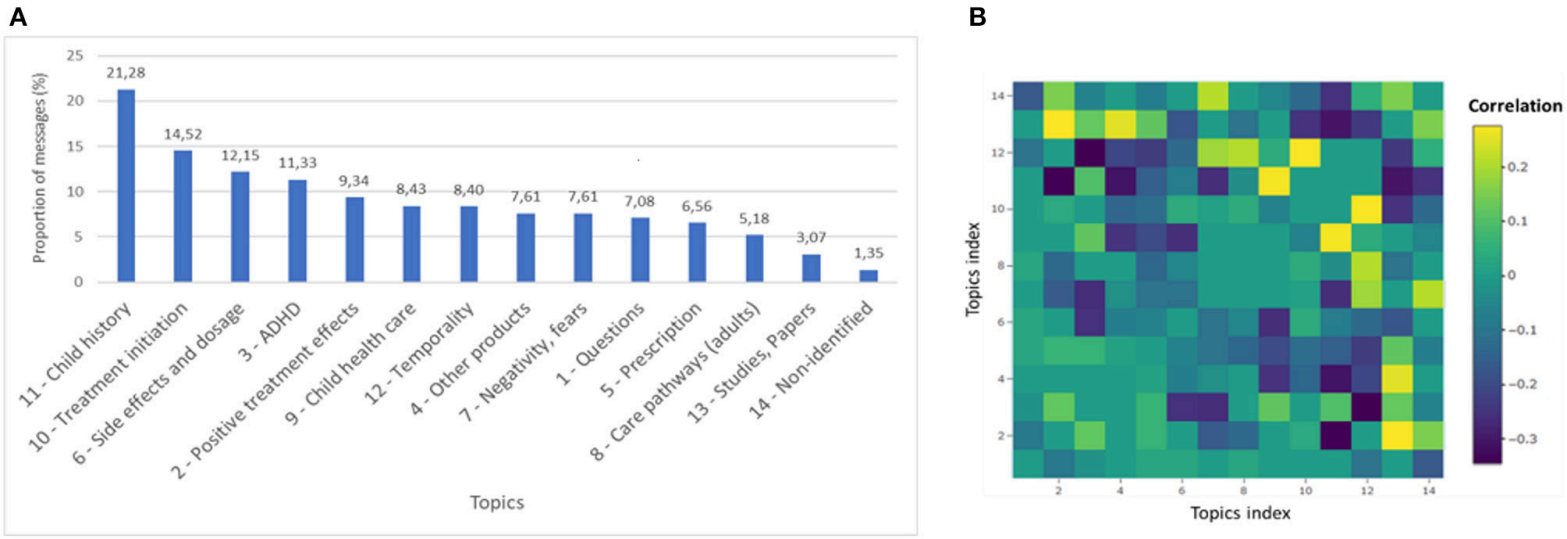
Topics description. **(A)** Messages distribution per topic. **(B)** Correlations between topics. Positive correlations are indicated in yellow. Negative correlations are indicated in blue.

A message was considered associated to a topic when it contained a proportion of words in the message associated to this topic superior to a threshold determined empirically (in our case 19%). The number of messages associated with each topic and the correlations between the topics are displayed in Figure 3.

The most represented topics were *Child history, Treatment initiation* and *Side effects and dosage*. The seven most frequent topics reflected usage that was consistent with the authorized indications of methylphenidate, even if there were some concerns with the product. Correlations were found within this list of seven topics, with associations between *Child health care* and *Child history*, as well as between *Temporality* and *Treatment initiation*, which seemed quite coherent.

Misuse could be identified in the topic named *Other products*, which included words like “*cocaine”* and “*sleeping pill”* and terms related with depression and anxiety (“*depression,” “Xanax*®”* and “Wellbutrin*®”). The topic *Negativity and Fears* contained words negatively connotated (“*fear,” “interruption”*) and words related to schizophrenia (“*schizophrenia,” “schizophrenic”)*. Use of methylphenidate by adults could also be identified through the topic *Care pathways for adults*.

#### Analysis of topics of interest

A Descending Hierarchical Classification (DHC) was performed on the topic associated with issues encountered while using the products in normal conditions (Topic 6—*Side effects and dosage*), on the topic related with use of methylphenidate by adults (Topic 8—*Care pathways for adults*), and on two topics that could be related to misuse (Topic 4—*Other products* and Topic 7—*Negativity and fears*).

##### Side effects (topic 6)

**Figure 4:**
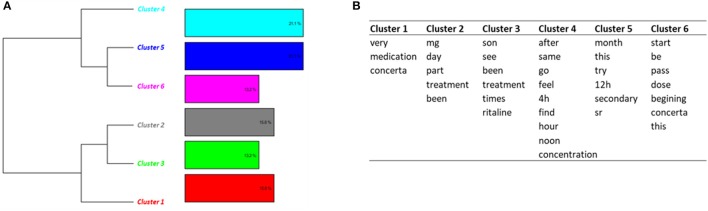
Results of the DHC applied on Topic 6—*Side effects and dosage*. **(A)** Dendrogram. **(B)** Words constituting each cluster.

Classification on Topic 6 identified 6 clusters (Figure 4). Most of messages (clusters 1, 2, 3, 4, 6) were related to the characteristics of the treatment, including dosage, effects, duration of the effects, initiation of the treatment. The messages in cluster 5 dealt with side effects, including loss of appetite, loss of weight, nausea, vomiting and fatigue.

##### Methylphenidate use by adults (topic 8)

**Figure 5:**
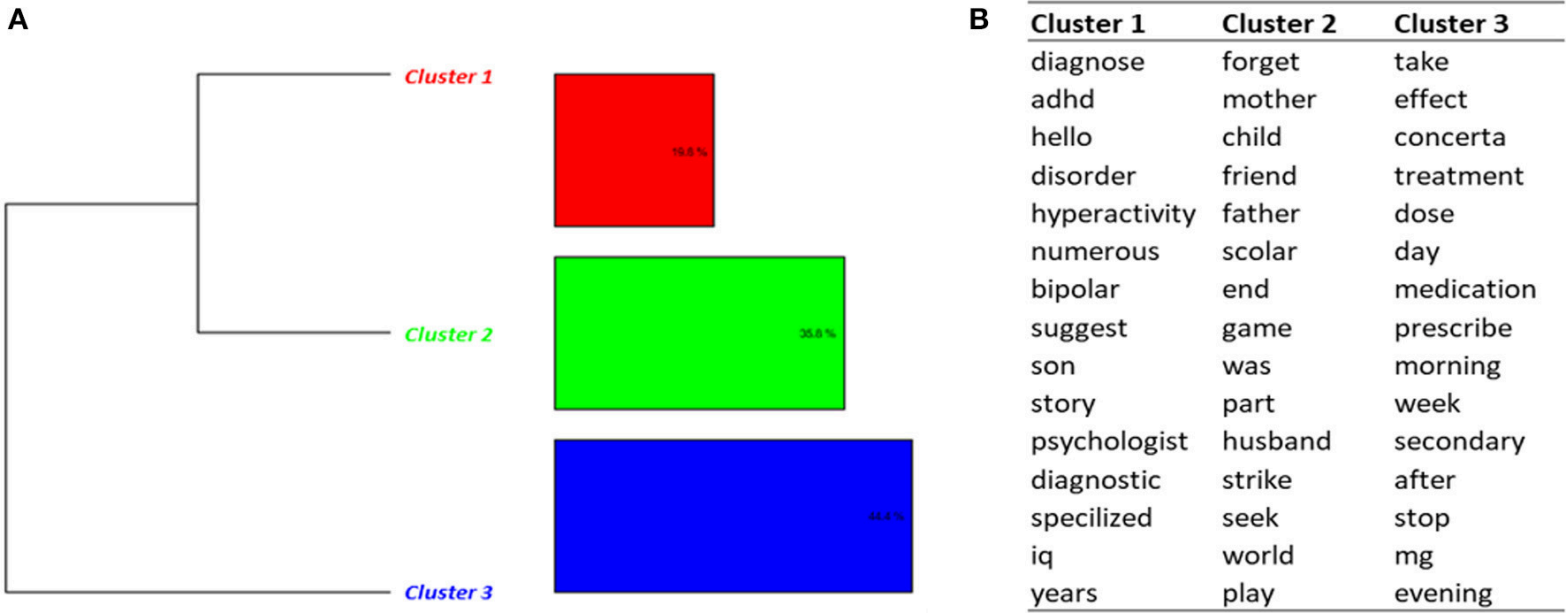
Results of the DHC applied on Topic 8—*Care pathways (adults)*. **(A)** Dendrogram. **(B)** Words constituting each cluster.

The DHC performed on Topic 8 (*Care pathways—Adults*) identified 3 clusters (Figure 5). Messages from adult patients diagnosed with ADHD were present in cluster 1. Some patients reported on very late diagnosis of ADHD, difficulties during childhood related to the absence of diagnosis at this time, and the positive effects of the treatment after the first intakes despite some negative effects. Several people reported difficulties to get an accurate diagnosis, confusion between ADHD and bipolarity, and difficulties to be tested when adults (cluster 2). In cluster 3, we found messages associated with dosage, treatment regimes, and cessation of treatment. One message was about methylphenidate for hypersomnia.

##### Misuse (topics 4 and 7)

**Figure 6:**
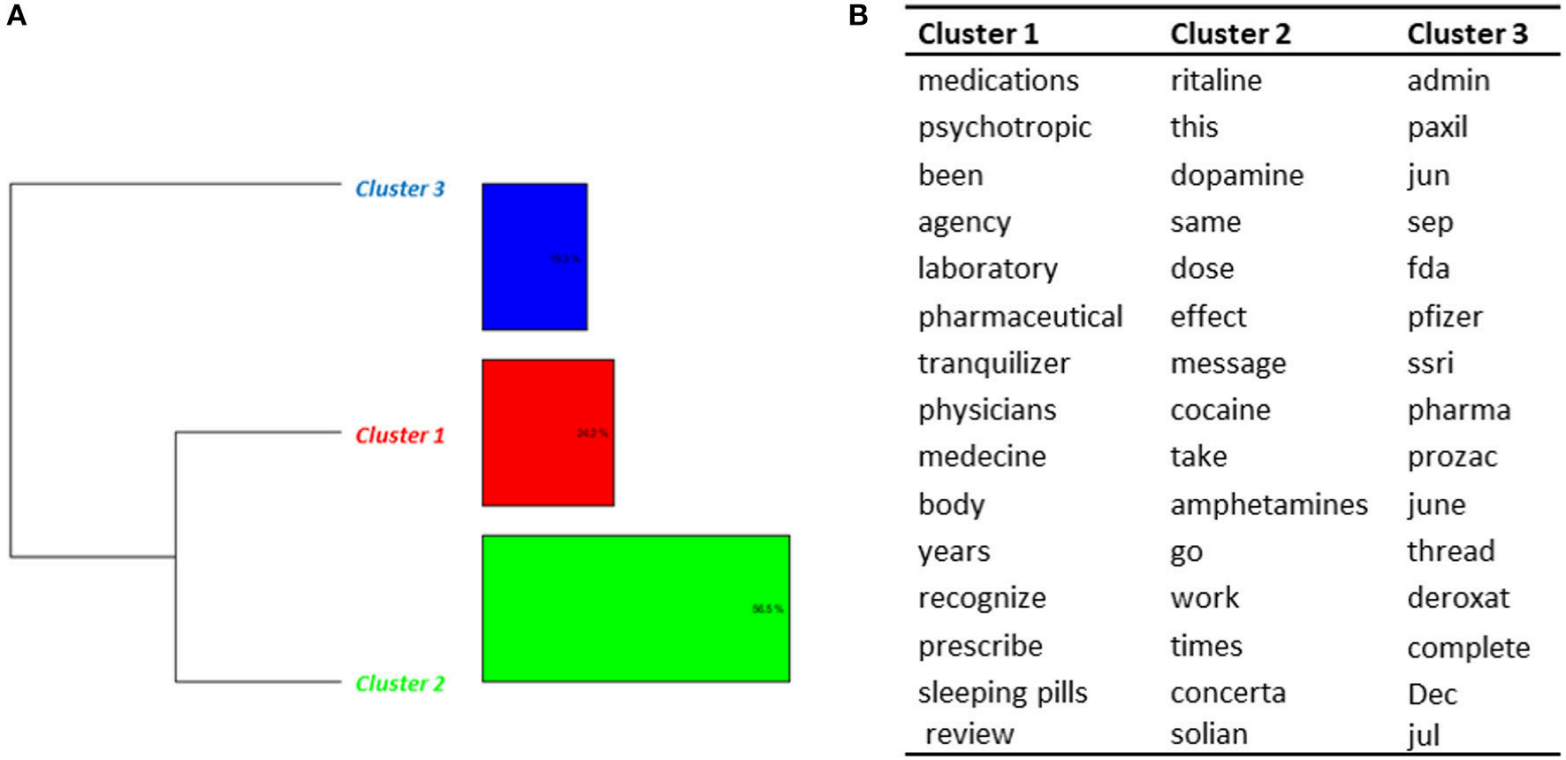
Results of the DHC applied on Topic 4—*Other products*. **(A)** Dendrogram. **(B)** Words constituting each cluster.

The DHC conducted to the identification of 3 clusters in Topic 4 (Figure 6). Most messages (clusters 1 and 3) were broad discussions about methylphenidate, and the potential dangers of taking it, as described in published articles. A comparison was made by some users between methylphenidate and other drugs (Paxil®, Prozac®, Deroxat®, Zoloft®). Part of this topic (cluster 2) dealt with the effects of methylphenidate and its similarities with amphetamines (some patients reported having tried both). Comparisons were also made with cocaine. Some cases of misuse of methylphenidate were identified. They consisted in parents taking the drug prescribed to their children to experiment its effects, students trying to enhance their abilities, and recreative effects.

**Figure 7:**
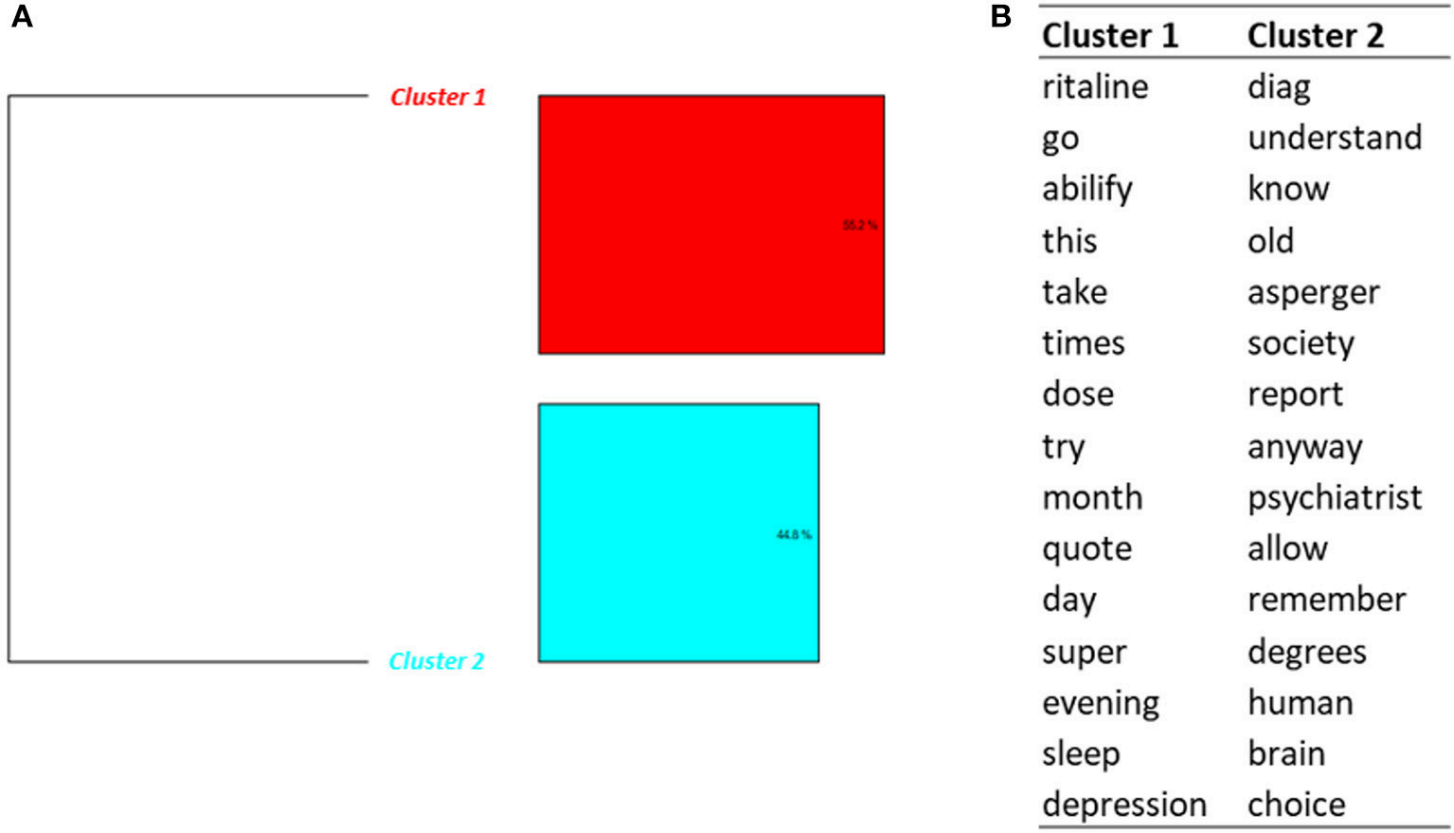
Results of the DHC applied on Topic 7—*Negativity and fears*. **(A)** Dendrogram. **(B)** Words constituting each cluster.

Classification on Topic 7 (*Negativity and fears*) conducted to 2 clusters (Figure 7). Some messages (cluster 1) were written by patients taking both an antipsychotic drug (Abilify®) and methylphenidate. Two messages were about quitting methylphenidate and Abilify®. In one message the patient considered that Abilify® had caused a disturbance in attention. One person decided to quit methylphenidate and to buy amphetamines instead.

Cases of misuse were also found, like one message about “snorting” methylphenidate while working.

Cluster 2 contained messages about methylphenidate and autism. For example, two messages were dealing with persons diagnosed Asperger and ADHD.

### Summary of the results

We automaticallyidentified 3443 posts about methylphenidate from the corpus of 21 millions messages published between 2007 and 2016, among which 61 adverse drug reactions (ADR) were automatically detected. Two pharmacovigilance experts evaluated manually the quality of automatic identification, and a f-measure of 0.57 was reached. Patient's reports were mainly neuro-psychiatric effects. Applying PRR, 67% of the ADRs were signals, including most of the neuro-psychiatric symptoms but also palpitations. Topic modeling showed that the most represented topics were related to *Childhood and Treatment initiation*, but also *Side effects*. Cases of misuse were also identified in this corpus, including recreational use and abuse.

## Discussion

### Pharmacovigilance and methylphenidate

In this study, text mining techniques were used to detect ADRs in social media with encouraging results. Starting with the overall corpus of messages containing drug names, the system was able to detect messages with potential ADRs. A manual review was however needed to exclude some false positives when the message was unclear. Moreover, our approach demonstrated its ability to detect phamacovigilance signals. 66.7% of the relationships involving methylphenidate extracted automatically from social media (26 out of 39) have been detected as a signal. Among these, 88.5% have been alarmed in VigiBase. An example of ADRs associated with methylphenidate that was detected as a signal from social media but not in VigiBase is muscle spasms. These results suggest that messages in forum could be used as an additional data source of knowledge for drug safety.

The topic analysis demonstrated that most topics in posts about methylphenidate were related to usage that was consistent with the marketing authorization. However, adverse events were a significant concern among the patients, corresponding to the third most discussed topic. Effects identified by topic modeling included psychiatric symptoms, effects on nervous system, loss of appetite, loss of weight, nausea, vomiting and fatigue. Methylphenidate use by specific populations like adults could be identified. Misuse was also a topic patients dealt with, including non-medical use and off-label use. Non-medical use that patients reported included parents testing methylphenidate prescribed to their children and students trying to enhance their abilities. Off-label use included methylphenidate use in patients suffering from psychosis. Cases of abuse could be identified too, e.g., using methylphenidate for recreative effects, snorting methylphenidate and replacing methylphenidate by amphetamines. Interestingly, some discussions stressed positive effects of the drug.

Further analysis could be done to identify pharmacovigilance issues related to methylphenidate. In this analysis, we compared the trend of messages across time with the key pharmacovigilance events related to methylphenidate, but it could interesting to compare the number of ADR across time to particular circumstances, like examination periods, as it has been presented by the EMA during the workshop on social media in 2016^11^. The presented analysis of 5000 tweets about methylphenidate and their trend comparison with examination periods yielded to the suggestion that misuse could be found in educational institutions at time of examinations, supporting our conclusions.

This work emphasizes the potential interest of monitoring adverse drug reactions on social media but there is still insufficient evidence to define how such monitoring should be integrated within the current pharmacovigilance process. The US Food and Drug Administration has published recommendations for the industry when using social media^12^ which describe how risk and benefit Information for drugs should be presented^13^ or the correction of independent third-party misinformation about drugs^14^, but has provided no guidance on the way social media should be monitored for pharmacovigilance signals.

The EMA recommends that “The marketing authorization holders should regularly screen the internet or digital media (web site, web page, blog, vlog, social network, internet forum, chat room, health portal, etc.) under their management or responsibility, for potential reports of suspected adverse reactions ^15^.” Therefore, it is not mandatory to monitor adverse drug reactions on digital media, which are not considered to be company sponsored.

The EMA is participating to the Innovative Medicines Initiative (IMI) Web-Recognizing Adverse Drug Reactions (RADR) project (Ghosh and Lewis, 2015). According to the report of the Work Package 1 Workshop, “It is likely that the social media utility will proof beneficial mainly for niche areas. It could also be tailored to the safety profile of a product or to be used as a source to answer specific regulatory questions where an aggregate review could be required^16^.” The current study showed that applying topic models to niche areas such as evaluation of abuse and misuse was efficient for investigating such issues with methylphenidate. It is desirable to investigate how social media could help regulatory authorities to explore other niche areas such as exposure during pregnancy or early monitoring of new products.

### Strengths

Determining which co-occurrences of drugs and symptoms in messages are true ADRs is a challenging task because of the complexity of modeling the linguistic pattern of causality. However, our study demonstrates the feasibility of the extraction of information on drugs and related ADRs from Web forums. We implemented a lexicon-based method to extract drug names and medical entities from posts. The system was based on the Smart Taxonomy Facilitator (STF) Skill Cartridge™ developed by Expert System. The system exhibits good NER performance, with F-measures of 0.94 and 0.81 for recognition of drugs and symptoms respectively (Chen et al., 2017). These results are similar to those obtained by other authors on messages in English (Lardon et al., 2015). Regarding French language social media, Morlane-Hondere et al. obtained a F-measure of 0.95 for chemicals, 0.86 for signs/symptoms and 0.82 for diseases using classifiers based on Conditional Random Fields and Support Vector Machines, which is also equivalent to our results (Morlane-Hondère et al., 2016). We retrieved in social media several signals that were found associated with methylphenidate in traditional data sources. We did not detect new ADR signals related to methylphenidate from our corpus. We plan to conduct a more extensive and systematic comparison of the ADRs on other drugs

Topic modeling approaches are rather new in medical domain, and most of the studies have focused on tweets rather than web forums. Other studies focusing on medical themes and forums messages like (Yang et al., 2015; Tapi Nzali et al., 2017) used the same LDA model. Two pharmacovigilance experts of our consortium (ALL and CB) performed an internal analysis of our results and concluded that this approach was a useful method that enhances expert' s ability to explore and analyze huge sets of text data. Above all, topic modeling performs automated annotation of such large datasets with latent “topic” information. We plan to conduct further evaluation with more experts in the future. Moreover, we showed that, besides ADRs, social media could be used to identify unexpected misuse behaviors—like parents taking pills prescribed to their child—that are impossible to detect from other sources.

### Limitations

A limitation of our study is inherent to the particularities of social network users who do not reflect the characteristics of patients population. This population bias was described by Ghosh and Guha (2013) for Twitter, and there is still an important lack of information regarding users' profiles. However, contrasting with tweets, the narratives that we focused on provided detailed information about patients' attitudes toward methylphenidate.

Although we obtained interesting performance regarding automatic identification of ADRs, the approach may be improved. Most of the false negatives were due to the constraint of sentence boundary of the cartridge, i.e., the automatic tool was asked to identify causal relationships between drug and symptom within the same sentence. Although the system was able to detect signals despite these false negatives, the current study showed that further work is needed to improve the power of our method. On the other hand, the false positives were due to (1) imprecise normalization of symptoms to MedDRA terms, (2) spelling and grammatical mistakes in colloquial expression.

In our dataset, the proportion of drug-event combinations of methylphenidate with one, two, three and four or more reports was 64.1, 17.9, 15.4, and 2.6% respectively, compared to 50.6, 27.8, 6.7, and 14.9% for all drug-event combinations, showing that methylphenidate is a less than average reported medication and that most of the ADRs are of very small frequencies, which makes it unsuitable to apply signal detection methods with criteria “3 or more cases” as decision rule. As our main objective was to illustrate with methylphenidate our methods of using text-mining tools for social media data, but not to compare different signal detection methods or to compare signals of methylphenidate with other drugs, we just listed the signals of methylphenidate with four most common used signal detection methods based on disproportionality, and did not explore all the signals in this work. However, these methods might be not absolutely appropriate for social media data, thus require further evaluation and potential adaptation and improvement.

Topic modeling also exhibits some limitations. Inherent to the topic model is the need that a human labels each topic, based on the list of characteristics words. Labeling of the topics by human brings subjectivity to this task (Ghosh and Guha, 2013). A solution to minimize this impact could be to perform double blind labeling of topics by two different experts. However, this step could be time-consuming in case of a huge number of topics, as described in another study (Tapi Nzali et al., 2017).

The sensitivity of topic models is rather low: very specific and sparse subjects would not be identified, as they are not discussed enough to generate a topic, as described by Prier et al. (2011). The precision of the model, however, is high.

Another limitation lies on the way topics are applied on words. As described, words are stemmed in order to be grouped together when they have common feature or pattern. However, stemming can conduct to group words with similar structure but different meanings. On the other hand, some words with similar meaning can have very different structures (for example the different forms of a conjugated verb in French). Lemmatization could be of help to overcome this issue.

### Complementarity of approaches

We developed two different approaches to analyze information automatically extracted from social media, signal detection and topic analysis. They provide complementary perspectives to understand the impact of a drug on patients. Signal detection allows to identify specific data related to possible new side effects. Topic models, on the other hand, provide an exploratory approach allowing to discover more qualitative information about the problematic related to drug use. Topic models allow to identify information nonspecifically investigated in the first place and to discover unexpected issues that can be more deeply investigated afterwards. In our study, we identified patients that presented psychiatric comorbidities (autism or schizophrenia) in addition to ADHD; we also identified posts where the patients compared the effect of methylphenidate to effects of amphetamines and cocaine, methylphenidate being called by these patients “low cost cocaine.” Contrasting with these messages, several patients or their relatives expressed fears about the dangers of methylphenidate and the possible addiction to the drug. These fears could be taken into consideration for example if we were to study non observance to methylphenidate. Another subject that could be analyzed more in-depth is adults having difficulties to get a diagnosis about hyperactivity and getting incorrect diagnosis. Advantages of using hypothesis-free models like topic models are their ability to highlight unknown issues that could benefit from further investigation and to provide health professional with further insights on patient behaviors.

## Author contributions

The two first authors, XC and CF contributed equally to the manuscript. XC, CF, and AB conceived the study. XC, CF, and AB designed the protocol, conducted the study, analyzed the results and drafted the manuscript. CF, PF, NT and SS developed the topic models. CH, SP and BD developed the NLP modules. XC, AG-A, PK, SK, and AB developed the signal detection module. CB and AL-L-L reviewed the messages and contributed to the analysis and evaluation steps. All authors discussed the results and contributed to the manuscript. All the authors were involved in the ADR-PRISM project (coordinator: NT).

### Conflict of interest statement

The Vidal drug database is owned by the Vidal Company, which employs SP. The Luxid Annotation server and the Skill Cartridge are owned by the Expert System Company, which employs CH. Kappa Santé, the company that developed the Detec't tool, employs CF, PF, SS, and NT. The other authors declare that the research was conducted in the absence of any commercial or financial relationships that could be construed as a potential conflict of interest.

## Footnotes

^1^https://www.fda.gov/ScienceResearch/SpecialTopics/RegulatoryScience/ucm452304.htm

^2^http://www.ema.europa.eu/ema/index.jsp?curl=pages/regulation/general/general_content_000258.jsp

^3^http://apps.who.int/medicinedocs/en/d/Js4893e/

^4^http://ansm.sante.fr/S-informer/Points-d-information-Points-d-information/Methylphenidate-donnees-d-utilisation-et-de-securite-d-emploi-en-France-Point-d-Information

^5^http://ansm.sante.fr/S-informer/Points-d-information-Points-d-information/Donnees-d-utilisation-et-mesures-visant-a-securiser-l-emploi-du-methylphenidate-en-France-publication-par-l-ANSM-d-un-rapport-d-analyse-et-d-une-brochure-d-information-a-destination-des-patients-et-de-leur-entourage-Point-d-information

^6^http://ansm.sante.fr/var/ansm_site/storage/original/application/fc636dd65bb327b11ceb1725e097bf6e.pdf

^7^https://www.whocc.no/atc/structure_and_principles/

^8^https://www.meddra.org/

^9^http://base-donnees-publique.medicaments.gouv.fr/

^10^https://www.who-umc.org/vigibase/vigibase/

^11^http://www.ema.europa.eu/docs/en_GB/document_library/Presentation/2016/11/WC500216438.pdf

^12^https://www.fda.gov/AboutFDA/CentersOffices/OfficeofMedicalProductsandTobacco/CDER/ucm397791.htm

^13^https://www.fda.gov/downloads/drugs/guidances/ucm401087.pdf

^14^https://www.fda.gov/downloads/drugs/guidances/ucm401079.pdf

^15^http://www.ema.europa.eu/docs/en_GB/document_library/Regulatory_and_procedural_guideline/2017/08/WC500232767.pdf

^16^http://www.ema.europa.eu/docs/en_GB/document_library/Report/2017/02/WC500221615.pdf
